# Pharmaceutical orientation at hospital discharge of transplant patients: strategy for patient safety

**DOI:** 10.1590/S1679-45082016AO3481

**Published:** 2016

**Authors:** Lívia Falcão Lima, Bruna Cristina Cardoso Martins, Francisco Roberto Pereira de Oliveira, Rafaela Michele de Andrade Cavalcante, Vanessa Pinto Magalhães, Paulo Yuri Milen Firmino, Liana Silveira Adriano, Adriano Monteiro da Silva, Maria Jose Nascimento Flor, Eugenie Desirée Rabelo Néri

**Affiliations:** 1 Hospital Universitário Walter Cantídio, Universidade Federal do Ceará, Fortaleza, CE, Brazil; 2 Universidade Federal do Ceará, Fortaleza, CE, Brazil

**Keywords:** Orientation, Patient discharge, Pharmacists, Kidney transplantation, Liver transplantation

## Abstract

**Objective:**

To describe and analyze the pharmaceutical orientation given at hospital discharge of transplant patients.

**Methods:**

This was a cross-sectional, descriptive and retrospective study that used records of orientation given by the clinical pharmacist in the inpatients unit of the Kidney and Liver Transplant Department, at *Hospital Universitário Walter Cantídio*, in the city of Fortaleza (CE), Brazil, from January to July, 2014. The following variables recorded at the Clinical Pharmacy Database were analyzed according to their significance and clinical outcomes: pharmaceutical orientation at hospital discharge, drug-related problems and negative outcomes associated with medication, and pharmaceutical interventions performed.

**Results:**

The first post-transplant hospital discharge involved the entire multidisciplinary team and the pharmacist was responsible for orienting about drug therapy. The mean hospital discharges/month with pharmaceutical orientation during the study period was 10.6±1.3, totaling 74 orientations. The prescribed drug therapy had a mean of 9.1±2.7 medications per patient. Fifty-nine drug-related problems were identified, in which 67.8% were related to non-prescription of medication needed, resulting in 89.8% of risk of negative outcomes associated with medications due to untreated health problems. The request for inclusion of drugs (66.1%) was the main intervention, and 49.2% of the medications had some action in the digestive tract or metabolism. All interventions were classified as appropriate, and 86.4% of them we able to prevent negative outcomes.

**Conclusion:**

Upon discharge of a transplanted patient, the orientation given by the clinical pharmacist together with the multidisciplinary team is important to avoid negative outcomes associated with drug therapy, assuring medication reconciliation and patient safety.

## INTRODUCTION

Patient safety corresponds to a reduction to a minimal level acceptable of risk of unnecessary damage associated with health care.^(1)^ Therefore, the issue of patient safety takes on particular importance in situations of transition of care, such as hospital discharge, since the use of medications at this point is complex, increasing the risk of medication errors due to incorrect or incomplete conveyance of information, besides involving multiple actions by multidisciplinary and inter-organizational teams.^(2,3)^

Hospital discharge is defined as a condition that allows patients to depart from hospital, encompassing all ways they may leave: as a result of a medical release, of the patient's own will, or as a result of death.^(4)^ In cases of medical release, it can characterize a time in which the patient evolves clinically and has the conditions required to return home and continue the recovery process.^(5)^ However, due to possible alterations in the drug treatment during transition of care (admission and discharge), the appearance of problems related to therapy arises, submitting the patient to harm soon after hospital discharge. In this way, early detection and minimization of adverse events, by means of orientation given to patients upon hospital discharge, contribute towards the success in continuity of treatment at home.^(6)^

The clinical pharmacists work together with the multidisciplinary team, preventing, detecting and resolving problems related to therapy, both during the hospital stay period and at discharge, since they have access to the patient, medical records, and sources of research.^(7)^ The work of the pharmacist at discharge may occur in different manners, such as medication reconciliation, identification of problems in compliance with treatment, orientation regarding several aspects of drug therapy, among others.^(8)^ All these activities are already provided by Resolution number 585 of the *Conselho Federal de Farmácia* [Federal Pharmacy Council],^(9)^ which regulates the clinical attributions of the pharmacist with the objective of promoting the rational use of medications and optimizing pharmacotherapy, aiming at defined results that improve patient's quality of life. The work of the pharmacist with the patient at hospital discharge decreases the differences between pre- and post-admission therapeutic regimes, improves compliance with treatment, reduces the appearance of drug-related adverse events, and diminishes the need for new hospitalizations.^(10)^ Chisholm et al. stated that a multidisciplinary approach in the post-transplant patient care including the clinical pharmacist is beneficial, especially for promoting compliance with treatment.^(11)^

Over the last years, international organizations have formalized the role and responsibilities of the pharmacist in the multidisciplinary team of transplant centers. Since then, clinical pharmacists have become necessary members of the transplant team, and they are responsible for the complete pharmaceutical assistance given to solid organ recipients.^(12)^

Immunosuppressant therapy is one of the types of care and routines that renal transplanted patients should follow, and it crucial for a successful transplant.^(13)^ Apart from complex immunosuppressive therapy, other drugs are prescribed, such as antivirals, antibiotics, antifungals, and medications for chronic diseases.^(14)^ The relation between the therapeutic complexity and the occurrence of favorable or undesirable clinical outcomes can collaborate towards optimization of the pharmacological treatments.^(15)^ The clinical pharmacist can evaluate the impact of the interventions on the clinical outcome, that is, the true results of interventions involving drug therapies.^(16)^

## OBJECTIVE

To describe and analyze the discharge orientation given by the pharmacist to patients submitted to renal and hepatic transplants as a strategy for patient safety.

## METHODS

This is a cross-sectional, descriptive, and retrospective study that analyzed the hospital discharge orientations given by the clinical pharmacist at the inpatient unit of the Kidney and Liver Transplant Department, at the *Hospital Universitário Walter Cantídio*, in the city of Fortaleza (CE), from January to July 2014. At the department evaluated, the clinical pharmacist in charge and the resident pharmacists oriented the patients at their first discharge after the transplant.

The variables analyzed in this study, such as indicators of the department, were as follows: number of admissions/month at the transplant unit; number of hospital discharges/month; number of pharmaceutical orientation at discharge/month; number of medications prescribed/patient. The medication-related problems (MRP) and pharmaceutical interventions (PI) made were also quantified. Moreover, an analysis of the clinical outcomes obtained with the PI applied was made. For this, the clinical outcomes were classified according to standardized nomenclature by the Clinical Pharmacy Department of the hospital as follows: “improved”, when the health problem associated with the medication improved after the PI; “not evaluated”, when the outcome of the health problem was not evaluated; and “prevented”, when the problem related to the medication was identified, but the patient did not present with a health problem, despite the risk, and the PI was performed with the objective of preventing the patient from experiencing the health problem. The outcomes were evaluated in the records of the kidney and liver transplant outpatient clinics, with each patient serving as his/her own control (baseline).

The significance of the PI was classified according to the method by Farré et al.^(17)^ Negative results related to medications, *i.e.,* situations in which the patient presented with a health problem associated with the medication, or risk situations in which the patient could develop a health problem associated with the medication, were classified as per the Third Consensus of Granada.^(18)^ Additionally, the records of the Medical Records and Statistics Service (SAME) of the hospital were used as sources of information, and the pharmacotherapeutic follow-up was done by the organization clinical pharmacist and the resident pharmacists at the transplant unit.

The data analysis period covers the beginning of the methodology to evaluate clinical outcomes after the PI by the Clinical Pharmacy of the hospital where the study was conducted. All records of discharge orientations in the database of the Clinical Pharmacy of the organization were evaluated. We excluded of the records that were incomplete and that precluded analysis.

The PI were analyzed and classified according to acceptability of the players involved: pharmacist – physician; pharmacist – pharmacy service; pharmacist – patient; pharmacist – multidisciplinary team. The medications that generated the interventions were classified as per the Anatomical Therapeutic Chemical.^(19)^

The data were tabulated and analyzed using the Epi Info™ version 3.5.1 program and the Statistic Package for Social Sciences (SPSS), version 20.0, for Windows. The data were represented descriptively on tables with means and standard deviation (SD) values for the numerical variables, and proportions for the categorical variables. To check heterogeneity in the proportions observed in the categorical variables, a statistical analysis was made, using the χ^2^ test with a significance value of p<0.05.

The project was approved by the Research Ethics Committee of the *Hospital Universitário Walter Cantídio*, with approval number: 894.794, CAAE: 36975414. 9.0000.5045.

## RESULTS

**Figure 1:**
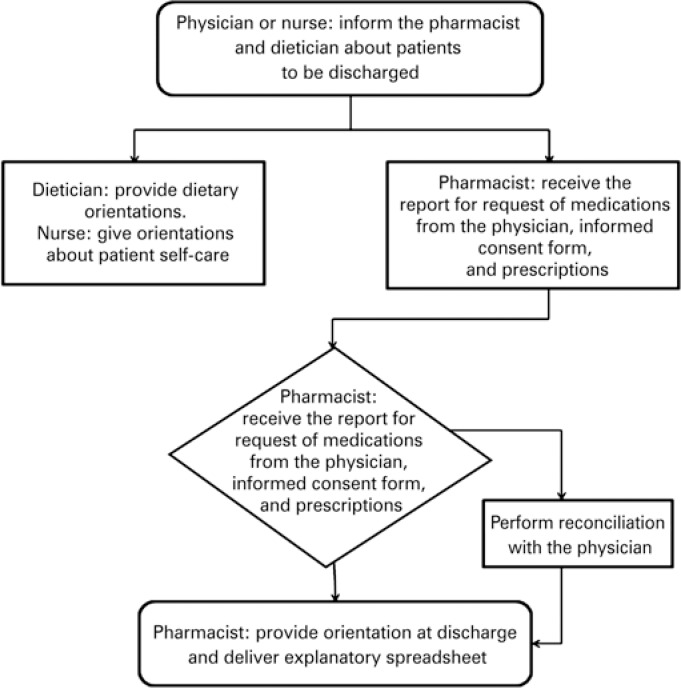
Flowchart of the orientation at the patient's first discharge after transplant

The first post-transplant discharge involved the entire multidisciplinary team, composed of physicians, nurse, dietician and pharmacist (Figure 1). All orientations given by the group to the patients were initiated by the medical analysis as to the patient's situation and the possibility of discharge, the nurses were responsible for instructing the patients about self-care, and the dietician for guiding about appropriate post-transplant diet. The clinical pharmacist of the department, along with the pharmacist residents, were responsible for the orientations regarding the prescribed drug treatment, such as the correct method of administration and storage of the medications, times of the doses, possibility of drug interactions or adverse reactions, information as to the process of availability by the Public Health System (SUS - *Sistema Único de Saúde*), besides highlighting the importance of compliance with treatment.

**Figure 2:**
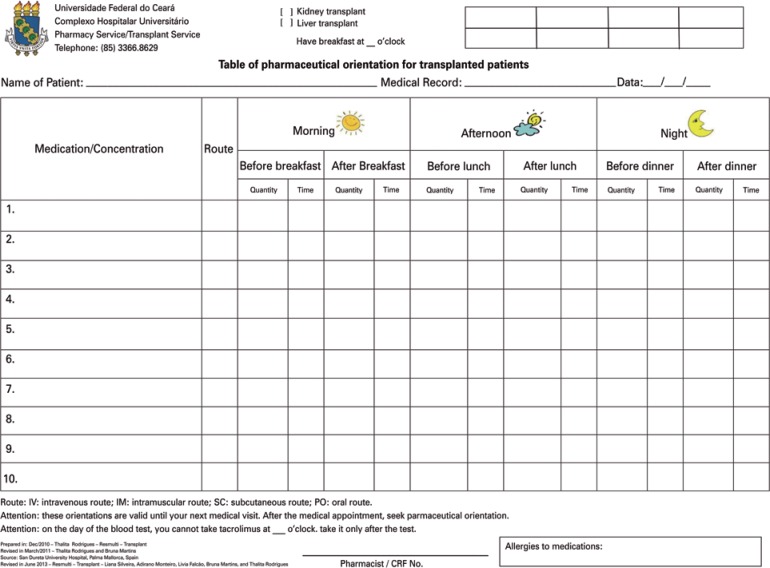
Table of pharmaceutical orientation given at patient's discharge

In the orientation given at the first post-transplant discharge, the patients received a personalized table with the medications prescribed by the physician, aiming to facilitate understanding of the drug treatment (Figure 2). The orientation is adjusted to the level of schooling and difficulty in understanding of the patient and caregiver.

During the period evaluated, 74 discharges were recorded of patients with kidney and/or liver transplants who received pharmaceutical orientation, accounting for a mean of approximately 10.6 (SD±1.3) discharges per month; 70.3% (n=52) of the patients instructed were male and 59.5% (n=44) had undergone liver transplantation. The patients that received orientations were prescribed a mean of 9.1 (SD±2.7) medications per patient. Specifically, the mean number of medications per patient and according to type of transplant was 7.5 (SD±1.7) medications for liver transplant and 11.5 (SD±2.4) for kidney transplants.

A total of 59 MRP were identified among the 74 oriented hospital discharges analyzed. The most frequent MRP were related to non-prescription of the medication needed at discharge (40;67.8%), associated with the dosage of the medications (subdose or overdose) (6;10.1%), test not requested (4;6.8%), absent or inadequate documentation for medication dispensing (3;5.1%), and unavailability of the medication (3;5.1%) (Table 1).

**Table 1:** Medication-related problems recorded at patient discharge

Medication-related problems	n (%)
Non-prescription of necessary medication	40 (67.8)
Subdose or overdose of the medication	6 (10.1)
Absent or inadequate documentation	3 (5.1)
Test not requested	4 (6.8)
Unavailability of the medication	3 (3.1)
Incorrectly written prescription	1 (1.7)
Prescription of unnecessary medication	1 (1.7)
Inadequate selection of medication	1 (1.7)
Total	59 (100)

In the analysis of the MRP, there were 18 different medications, and these were considered as per the Anatomical Therapeutic Chemical classification, according to which 49.2% (n=29) of the MRP cases involved medications with action on the digestive system and metabolism; 20.3% (n=12) were from the class of general anti-infectious drugs for systemic use, and 16.9% (n=10) acted in the blood and hematopoietic organs (Table 2).

**Table 2:** Medications involved in the pharmaceutical interventions performed at patient discharge

A - Digestive tract and metabolism: 49. 2% (n=29/59)	
Omeprazole	n=5
Prednisone	n=1
Nystatin	n=14
Pyridoxine	n=3
Insulin	n=3
Magnesium sulfate	n=1
Potassium chloride	n=2
J - General anti-infectious drugs for systemic use: 20.3% (n=12/59)	
Trimethoprim-sulfamethoxazole	n=3
Isoniazid	n=1
Valganciclovir	n=5
Ganciclovir	n=3
L - Antineoplastic agents and immunomodulators: 8.5% (n=5/59)	
Tacrolimus	n=4
Sodium mycophenolate	n=1
B - Blood and hematopoietic organs: 16.9% (n=10/59)	
Epoetin alpha	n=9
Enoxaparin	n=1
C - Cardiovascular system: 3.4% (n=2/59)	
Doxazosin	n=1
Propranolol	n=1
N - Nervous system: 1.7 (n=1/59)	
Fluoxetine	n=1

As to analysis of occurrence of negative outcomes associated with medications, the most frequent category was untreated health problem (53;89.8%), followed by quantitative insecurity (3;5.1%), quantitative ineffectiveness (2;3.4%), and the effect of unnecessary medication (1;1.7%).

Based on the identification of these problems, PI were conducted aiming at resolution/prevention of each one; in that, 54.2% (n=32) were in liver transplants and 45.8% (n=27) in kidney transplants. The request for inclusion of the medication was the predominant PI (39;66.1%), followed by the request for adjusting the dose of medication (6;10.2%) (Table 3).

**Table 3:** Classification of the pharmaceutical interventions conducted at patient discharge

Pharmaceutical interventions	n (%)
Request for inclusion of medication	39 (66.1)
Request for adjusting the dose of medication	6 (10.2)
Adjustment to the process of drug dispensing	6 (10.2)
Request for tests	4 (6.7)
Acquisition of health-related product	1 (1.7)
Request for correction of the prescription text	1 (1.7)
Request for withdrawing the medication	1 (1.7)
Request for replacing the medication	1 (1.7)
Total	59 (100)

All PI were accepted, and in 98.3% (n=58) of them the physician was the professional contacted. As to the significance of the PI, all were classified as “appropriate”, since they enhanced quality of care and/or treatment, increasing effectiveness or decreasing toxicity.

Analyzing the results of the interventions involving drug therapies, that is, the clinical outcome, we noted that 86.4% (n=51) were classified as “prevented”, since the PI conducted prevented the patient from having the health problem. It was observed that the different outcomes of the PI were heterogeneous among themselves, with a statistical difference among the observations (Table 4).

**Table 4:** Clinical outcomes after pharmaceutical interventions conducted at discharge from the patient

Clinical outcome	n (%)	p value
Prevented	51 (86.4)	
Improved	5 (8.5)	p<0.05*
Not evaluated	3 (5.1)	
Total	59 (100.0)	

* χ^2^ test for heterogeneity.

## DISCUSSION

Hospital discharge of transplanted patients is a process that involves the complete multidisciplinary team. The pharmacist is responsible for orienting about the drug treatment^(20)^ and should draw up a discharge plan, taking into consideration the particularities of the patient, so that the planning be appropriate for each individual case. Upon orientation, the pieces of information should not only be given verbally, since this may be insufficient for full understanding of the treatment prescribed. Therefore, the pharmacist should use instruments that enable direct contact with the patients and ease their understanding, such as using symbols, colors or figures that can illustrate what is described.^(21)^ With the purpose of individualizing the instructions according to the patient's comprehension level, the orientation table prepared by the clinical pharmacist is used.

In the analysis presented, there was a difference between hospital discharge at the transplant unit, and the number of discharges oriented by the pharmacist. This was due to the fact that the multidisciplinary team provides orientations at the first post-transplant discharge, but the unit where the project was carried out receives not only the newly transplanted patients, but also admits those with intercurrent events, regardless of when the transplant was performed. After the first pharmaceutical orientation at discharge, the patient is followed up by the multidisciplinary team in outpatient clinic visits. The studies showed that some activities, such as medication reconciliation, orientation to the patient and/or caregiver, and follow-up at patient´s home are strategies that minimize the risks of medication-related adverse events after hospital discharge, contributing towards avoiding damage to patients and unnecessary costs for the healthcare system.^(3)^

Apart from immunosuppressive therapy after transplant, bacterial, fungal or viral prophylaxis is required, as well as treating any comorbidities the patient might suffer from.^(14)^ However, considering the immunosuppressive and prophylactic therapies prescribed for transplants, there are more medications for kidney than liver transplants. This is due to differences in hospital protocols, in which there is dual standard maintenance immunosuppression for hepatic transplant (calcineurin inhibitor and corticosteroid), and triple regime for renal transplant (calcineurin inhibitor, antiproliferative agent, and corticosteroid). Furthermore, prophylactic therapy also demands more medications for immunosuppression.^(22)^

Most of the MRP in the discharge process were associated with drugs for prophylaxis (nystatin, omeprazole, and valganciclovir, for example) taken after transplant. Thus, it is possible to note that, at the transition from hospital care to discharge, there may be discrepancies in the prescribed drug regime that might cause adverse events.^(2)^ In addition, non-prescription of the medication necessary at discharge was also frequent, justifying more than half the PI related to requests for inclusion of medications. In this way, the pharmacist should participate in prescription of medications before providing orientations to patient at discharge, by means of medication reconciliation, helping the prescribing physicians and assuring correct drug therapy.^(23)^

Based on the PI conducted, in most cases the outcome was “prevented”, reducing the possibility of adverse events and assuring patient safety. The evaluation of the clinical outcome was possible by analyzing the patient's tests at discharge, as well as possible health problems that might occur if the MRP were to persist. The pharmaceutical orientation given to the patient at discharge aims to guarantee comprehension of the new therapeutic scheme; to treat the health conditions and prevent new problems; to explain to the patient about the importance of treatment and access to care; and consequently, to contribute towards optimizing compliance.^(24)^ Some studies showed that 6 to 12% of medication-related adverse events resulted in patients going to emergency departments, and 5% in hospital readmission.^(10)^

The literature is on this topic is scarce and the present study had the limitation of comparing the clinical outcome after PI in transplanted patients. There were no records in some periods of the study, which limited sampling.

## CONCLUSION

Hospital discharge of transplanted patients is a time when the clinical pharmacist, together with the multidisciplinary team, can orient patients about the mediactions prescribed, and solve and/or prevent the negative results associated with drug therapy. Additionally, it is necessary to assess the clinical outcomes after pharmaceutical intervention to measure the true results of these interventions and to assure patient safety.
